# Andrographolide Suppress Tumor Growth by Inhibiting TLR4/NF-κB Signaling Activation in Insulinoma: Erratum

**DOI:** 10.7150/ijbs.49433

**Published:** 2020-06-25

**Authors:** Qian-Qian Zhang, Yi Ding, Yan Lei, Cui-Ling Qi, Xiao-Dong He, Tian Lan, Jiang-Chao Li, Ping Gong, Xuesong Yang, Jian-Guo Geng, Li-Jing Wang

**Affiliations:** 1 Vascular Biology Research Institute, Guangdong Key Laboratory of Pharmaceutical Bioactive Substances, Guangdong Pharmaceutical University, Guangzhou 510006, China.; 2 Department of Biologic and Materials Sciences, University of Michigan School of Dentistry, Ann Arbor, MI 48109, USA.; 3 Department of Histology and Embryology, Key Laboratory for Regenerative Medicine of the Ministry of Education, Medical College of Jinan University, Guangzhou 510632, China.

In our paper 1, Due to the authors made an error p50 IHC figure in Figure 4B and error TLR4, MyD88 and p65 IHC figures in Figure 5B. The authors would like to apologize for any inconvenience caused to the readers by these changes. Through repeated confirmation, we confirmed that we attached the wrong IHC figures due to mislabeling the storage photos while taking photos. We redone the IHC assay and confirmed that the results were in accordance with the published results. It is important to state that this correction do not affect our study's results in the published paper. The 40× and 100× original figures of p50 during tumor development and TLR4, MyD88 and p65 in Mock, DMSO- or Andro-treated groups were attached as supplementary figures. I would like to declare on behalf of my co-authors that all authors have seen and approved the Erratum being submitted. Figure 4 and Figure 5 should be corrected as follows.

## Figures and Tables

**Figure 4:**
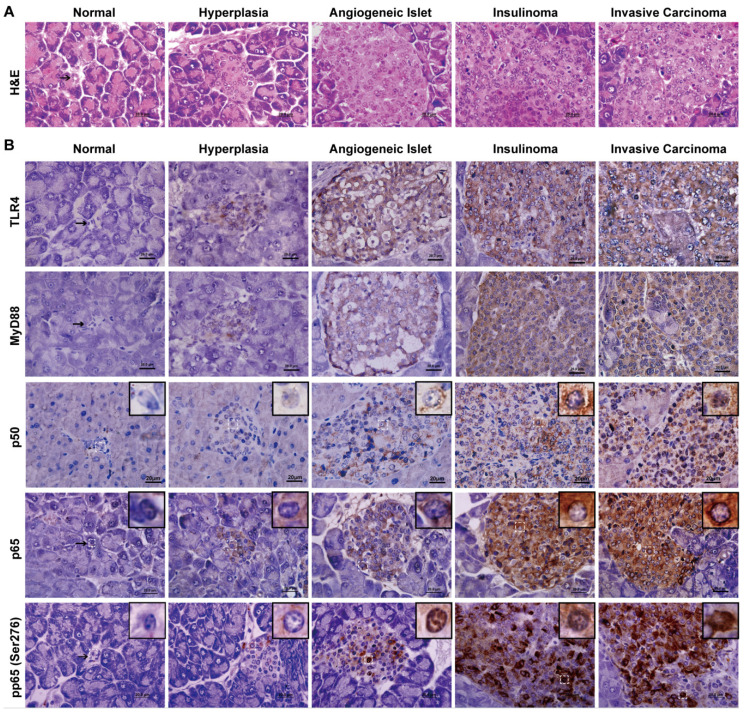
The expression and activation of TLR4/NF-κB signaling is increased during insulinoma development. (A) The classification of insulinoma. The H&E staining defined the stages of insulinoma. (B) The immunohistochemical staining of TLR4, MyD88, p50, p65 and phosphorylated p65 (Ser276) at the stage of normal, hyperplasia, angiogenic islet, insulinoma, and invasive carcinoma in RIP1-Tag2 mice. Results are representative of at least 3 tissue samples in a mouse from more than 3 mice for each stage. Bar, 20 μm.

**Figure 5:**
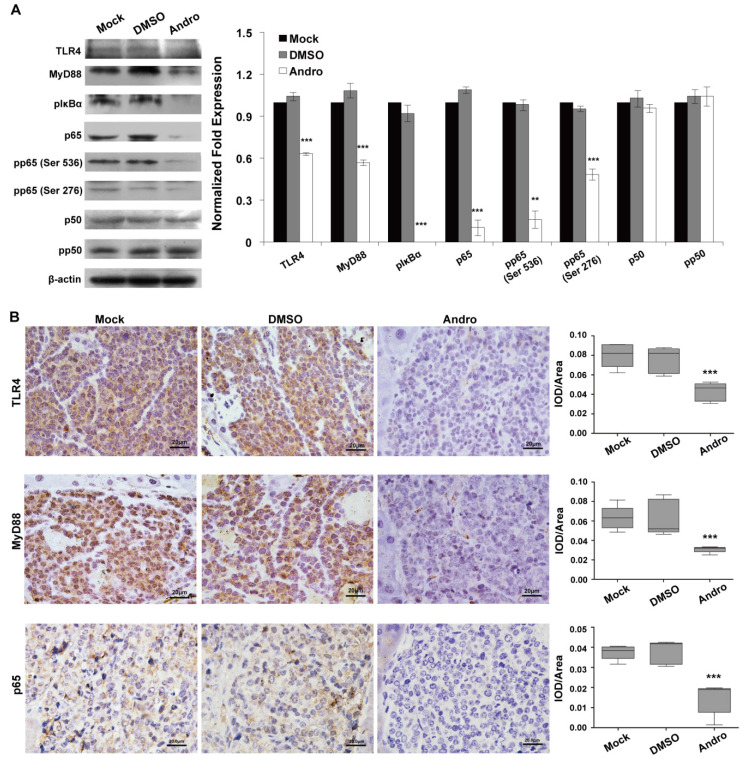
Andro targets TLR4/NF-κB signaling in insulinoma. Andro can significantly inhibited the protein expression of TLR4, MyD88, phosphorylated IκBα, p65, phosphorylated p65 (Ser536), and phosphorylated p65 (Ser276) of TLR4/NF-κB signaling pathway in tumor tissues of RIP1-Tag2 mice compared with control groups. Immunohistochemical staining showed that Andro also inhibited the expression of TLR4 (B), MyD88 (C) and p65 (D) in tumor tissues of RIP1-Tag2 mice compared with control groups. Bar, 20 μm. ***P < 0.001.
